# Jagged1 and Epidermal Growth Factor Promoted Androgen-Suppressed Mouse Hair Growth *In Vitro* and * In Vivo*

**DOI:** 10.3389/fphar.2019.01634

**Published:** 2020-01-31

**Authors:** Yufeng Lin, Canying Liu, Xiaoshu Zhan, Bingyun Wang, Kui Li, Julang Li

**Affiliations:** ^1^ Department of Life Science and Engineering, Foshan University, Foshan, China; ^2^ Department of Animal and Poultry Science, University of Guelph, Guelph, ON, Canada; ^3^ Institute of Animal Science, Chinese Academy of Agricultural Sciences, Beijing, China

**Keywords:** Jagged1, epidermal growth factor, hair growth, hair follicle, testosterone

## Abstract

Recent studies have reported that T-reg cells are intimately linked with hair follicles in a stage-dependent manner and play an important role in hair follicle cycling and regeneration in murine skin. Further study revealed that T-reg cell’s regulation of hair follicle growth is through its preferential expression of the Notch ligand Jagged‐1 (Jag1), which facilitates hair follicle regeneration. However, the role of Jag1 in androgen-suppressed hair growth is yet to be investigated. In addition, although epidermal growth factor (EGF) is a mitogen for cells including skin cells, whether it works synergistically with Jag1 to enhance hair follicle development is unknown. The current study intended to investigate effects of topical application of Jag1 on androgen-suppressed hair growth, and to determine the potential synergistic effect of EGF and Jag1 in this process *in vivo*. Fifty mice were depilated at the dorsal back area to achieve synchronized anagen development, and randomly divided into five groups with the following topical treatments control for 14 days; testosterone to induce androgenetic alopecia; Jagged1 (testosterone + Jagged1); EGF (testosterone + EGF); and Jagged1 + EGF (testosterone + Jagged1 + EGF). It was found that EGF and Jag1 by itself respectively, did not promote androgen-suppressed hair growth significantly. This stimulating effect was enhanced in the presence of both EGF and Jagged1 (p < 0.05). The hair growth promoting effect was accompanied by better follicle growth, which is associated with increased cell proliferation in the hair follicle and altered the expression of genes that are important in hair follicular cell proliferation and differentiation. Our results provide insights into the therapeutic potential of these peptides for androgenetic alopecia.

## Introduction

Androgenetic alopecia (AGA) is the most common cause of hair loss in men. It is an androgen-dependent dermatological disorder, in which an alteration in the hair cycle dynamics leads to vellus transformation of terminal hair follicles (Kaliyadan et al., 2013). Due to the limited treatment options, searching for alternative treatment therapeutics in AGA has been a continuous mission for scientists in the field.

The hair follicle undergoes cycles of renewal in three phases known as anagen, catagen, and telogen. Each new cycle enables the development of the hair follicle from an active hair shaft production stage (anagen) to quick involution (catagen) and finally, to resting (telogen) stages. The development of these phases involves intensive organ remodeling, especially during the anagen phase when the growing follicle migrates downwards through the dermis, and during the catagen phase when involution of the hair bulb takes place (Zhang et al., 2016a). Skin is one of the largest organs and is home to a large proportion of immune cells. Foxp3^+^ CD4^+^ regulatory T (T-reg) cells are a subset of immune cells that function to regulate tissue inflammation. A recent study by Ali et al., has revealed that T-reg cells in murine skin are intimately linked with hair follicles in a stage dependent manner during follicular cycling (Ali et al., 2017). In skin tissue, there is a population of stem cell that resides in the bulge region of hair follicles (HFSCs). Dysregulation of HFSC can result in stem cell inactivation and lack of hair follicle development, leading to loss of hair regeneration. The same research group further showed that T-reg cells play a role in hair follicle cycling and hair regeneration in murine skin. Moreover, it was found that T-reg cell’s regulation of hair follicle growth is through its preferential expression of high levels of the Notch ligand, Jagged‐1. Jagged1 stimulates HFSC proliferation and differentiation, thus facilitating hair follicle regeneration (Ali et al., 2017). However, the role of Jagged1 in androgen-suppressed hair regrowth has not been explored.

Epidermal growth factor (EGF) is widely expressed in skin, although the effect of EGF on the development and growth of hair follicle has been under debate. During early development when skin tissues were isolated from 12.5 or 13.5 days postcoitus embryos, EGF was found to inhibit hair follicle formation (Kashiwagi et al., 1997). However, topical application of tyrphostin AG1478, a specific inhibitor of EGFR, completely inhibited new hair growth in adult mice, suggesting that EGF receptor activation is indispensable for the initiation of hair growth (Mak and Chan, 2003). In addition, EGF promoted the growth and migration of mink hair follicle outer root sheath (ORS) cells *in vitro* (Zhang et al., 2016b). Using culture wool follicles from sheep, it was found that the bulb cell population was induced to differentiate into an ORS phenotype by EGF (Bond et al., 1996). These findings suggest that the regulation of EGF on hair follicle growth may be stage and cell type specific. It appears that its role in adult animals is stimulatory, and targets the outer root sheath cell growth and bulb cell differentiation more. It is thus conceivable that other regulator(s) such as Jagged1, which targets hair follicle stem cells within the inner root more, are required to collaboratively and effectively promote hair follicle growth.

The objectives of the study were 1) to investigate the effect of topical application of Jagged1 on hair growth, and 2) to determine the potential synergistic effect of EGF and Jagged1 in a androgen-suppressed hair regrowth model *in vivo*. We found that the EGF and Jagged1 enhanced each other’s hair grow promoting effect, which was accompanied by better follicle growth. This is associated with increased cell proliferation in the hair follicle and altering the expression of genes that are important in hair follicular cell proliferation and differentiation.

## Materials And Methods

### Animals

Eight-week-old male C57BL/6 mice were obtained from Guangdong Medical Laboratory Animal Center (Guangzhou, China), and then allowed to adapt for a week with food and water *ad libitum*. Mice were cared under 26 ± 2°C room temperature and 12 hours of a light/dark cycle. This study had been reviewed and approved by the Foshan University, Institutional Animal Care and Use Committee (FSUeae2018014).

### Hair Growth Study

An androgenetic alopecia model was used with modifications (Truong et al., 2017; Zhang et al., 2018). Fifty mice at 9 weeks of age were used as the animal model to determine the effect of Jagged1 and/or EGF on androgenetic alopecia. After adapting to the environment, mice were anesthetized with 200 µl of 4% chloralhydrate intraperitoneally, and the dorsal area (2 × 2 cm) was shaved with an animal clipper and applied with hair removal cream externally to allow hair follicle synchronization. The dorsal depilatory mice were randomly assigned to five treatment groups. As testosterone was dissolved in 30% (v/v) ethanol, the control group received 30% ethanol on the depilation area alone; the testosterone group received 0.5% testosterone (Patel et al., 2014); the Jagged1 group received 0.5% testosterone followed by 1 mg/ml of Jagged1 one hour later; the EGF group received 0.5% testosterone followed by 30 µg/ml of EGF 1 hour later; the Jagged1 and EGF group received 0.5% testosterone followed by 1 mg/ml of Jagged1 and 30 µg/ml of EGF 1 hour later. Synthesized Jagged1 (CDDYYYGFGCNKFCRPR; Shanghai Top-peptide Biotechnology Co., Ltd.) and EGF (Levesque et al., 2018) were dissolved in sterile water to a final concentration of 1 mg/ml and 30 µg/ml, respectively. Testosterone (Dalian Meilun Biotechnology Co., Ltd; No: MB1636) was dissolved in 30% ethanol to a final concentration of 5 μg/ml. From day 1 after hair removal, each compound (100 µl) was topically applied to the shaved dorsal area once a day for 7 or 14 days. Mice in the control group were firstly applied with 100 µl of 30% alcohol. After 1 h, 100 µl of sterile water was applied. The testosterone group and the treatment group were applied with 100 µl of 0.5% testosterone, followed by the sterile water and peptide(s) as indicated above 1 h later, respectively.

To examine the hair growth promoting effect, the darkening of mice skin color was observed every day. At day 0, 7, and 14 after topical application, the mice were photographed. Hair re-growth efficacy score was measured as 0, 1, 2, 3, 4, and 5 in correspondence to 0%, 20%, 20–40%, 40–60%, 60–80%, 80–100% of hair growth, respectively.

#### Isolation and Culture of Mouse Hair Follicles In Vitro

The procedure was performed following a published protocol (Tobin, 2011). After hair shearing was performed, hair follicles were isolated from the upper lip pad of 8-week-old C57BL/6 male mice under the binocular dissecting microscope (Shanghai optical instrument factory). They were randomly divided into the following groups: control (medium); Testo (20 μg/ml); Jagged1 (20 μg/ml Testo + 40 μg/ml Jagged1); EGF (20 μg/ml Testo + 1 ng/ml EGF), and Jagged1 + EGF (20 μg/ml Testo + 40 μg/ml Jagged1 + 1 ng/ml EGF). The groups were cultured in 48-well dishes for 36 h in Williams E medium (Gibco BRL, Grand Island, NY, USA, No:22551089) supplemented with 10 ng/ml hydrocortisone (Shanghai yuanye Bio-Technology Co., Ltd;No:S31360), 10 mg/ml insulin (Dalian Meilun Biotechnology Co., Ltd; No: MB5980), 10 mg/ml transferrin (Dalian Meilun Biotechnology Co., Ltd; No: MB3249), 2 mM L-glutamine, and 100 U/ml streptomycin (Gibco BRL, Grand Island, NY, USA, No:15140-122) at 37℃ in a 5% (v/v) CO_2_ atmosphere. The hair shaft elongation was measured at 0 and 36 h, respectively.

### Histological Analysis of Hair Follicles

Five mice of each group were euthanized on day 7 and 14 with diethyl ether, respectively, and skin tissue was extracted from the shaved dorsal area for tissue and following gene expression analysis. The dorsal skin samples were fixed in 4% formaldehyde for 24 hours and then processed by paraffin block embedding using standard techniques. General histology was visualized by H&E staining, and the dermis thickness was subsequently observed. Then, the number of hair follicles was determined under a microscope (Nikon, ECLIPSE E200).

### Immunohistochemical Examination

In order to describe Ki67-positive hair matrix cells in mouse hair follicles, immunohistochemistry was performed on 5-µm-thick, paraffin-embedded skin tissue sections. The sections were deparaffinized in xylene, and rehydrated by a series of graded ethanol rinses. After antigen retrieval in sodium citrate buffer, endogenous peroxidase activity was blocked with 10% bovine serum for 2 hours, followed by incubation overnight at 4°C with rabbit anti-Ki67 (Abcam, Cambridge, UK; No.: ab15580, 1:500) primary antibody. Subsequently, horseradish peroxidase conjugated secondary antibody (Service Bio, GB23303, 1:200) was pipetted on the sections and incubated for 30 minutes at 37℃. The negative control was subjected to the same steps as described above, but the primary antibody was replaced by 10% bovine serum. With diaminobenzidine (DAB), the reaction product was visualized as brown staining. Then, the examined sections were counterstained by hematoxylin. The expression level of Ki67 in the tissue sections were assessed and quantified using an Open Source Plugin based on the staining intensity, and then assigned a score as high positive (3+), positive (2+), low positive (1+), and negative (0) (Varghese et al., 2014).

### Terminal Deoxynucleotidyl Transferase dUTP Nick End Labeling (TUNEL) Assay

To measure apoptotic cells in mouse hair follicles, terminal deoxynucleotidyl transferase-mediated digoxigenin-dNTP nick end-labeling (TUNEL) staining was performed following the recommendations of the manufacturer (*In Situ* Cell Death Detection, POD Kit (Roche,11684817910)). Deparaffinized skin tissue sections were randomly taken from each group and treated with 20 μg/ml proteinase K for 25 min at 37℃ to strip proteins from nuclei, and then rinsed three times with PBS (pH = 7.4). Inactivation of endogenous peroxidase was performed by incubating with 3% H_2_O_2_ for 15 min. After hematoxylin counterstaining for 3 min, the normal cell nuclei were stained blue, and the TUNEL positive cell revealed by DAB were brownish yellow. All sections were examined immediately after the reaction and photographed with microscope (Nikon, ECLIPSE E200). For quantitative analyses, the number of TUNEL positive or Ki-67 positive cells was counted by using ImageJ software.

### RNA Extraction and RT-qPCR Analysis

The extracted skin tissues in the shaved dorsal area were collected and stored at −80°C for RNA analysis. Total RNA was isolated from the skin samples with RNAzol^®^ RT (Molecular Research Center, Inc. Cincinnati, OH; No.: RN 190) in accordance with the protocol. RNA was quantitated using a NanoPhotometer-NP80 (Implen, Germany). Eight hundred nanograms of total RNA of each sample was reversely transcribed into complementary DNA using PrimeScript™ RT reagent Kit with gDNA Eraser (TakaRa, Beijing, China; NO.: RR047A) and conducted in Biometra T professional gradient Thermocycler (Biometra, Germany). Quantitative PCR was performed using SYBR^®^ Premix Ex Taq™ II (Tli RNaseH Plus) (Takara, Beijing, China; No.: RR820A). Thermal cycling was performed for 30 seconds at 95°C for enzyme activation, denaturation for 5 seconds at 95°C, and annealing for 30 seconds at 60°C. The real-time PCR was performed for at least 40 cycles. A dissociation curve was generated to assure the absence of non-specific products or primer dimers. All primer sets used in this study were obtained from Sangon Biotech Co., Ltd. Primers (Shanghai) and expected product size are shown in **Table 1**. Product sizes were verified by agarose gel electrophoresis. Relative quantification was conducted with the 2^−∆∆Ct^ method (Livak and Schmittgen, 2001). Expression data of the genes of interest were normalized with the housekeeping gene β-actin. All real-time PCRs were performed in triplicate, and the changes in gene expression were reported as fold-increases relative to testosterone group.

**Table 1:** Specific sequences of the forward and reverse primers used in the RT-qPCR

**Gene**	**Sequence (5′→3′)**	**Size (bp)**	**NCBI Reference sequence**
TNF-α	F:GCCTCTTCTCATTCCTGCTTG R:CTGATGAGAGGGAGGCCATT	115	XM_021149738.1
Icos	F:ATGAAGCCGTACTTCTGCCG R:CGCATTTTTAACTGCTGGACAG	161	XM_006496139.1
FGF-7	F:TGGGCACTATATCTCTAGCTTGC R:GGGTGCGACAGAACAGTCT	147	XM_021156378.1
Shh	F:AAAGCTGACCCCTTTAGCCTA R:TTCGGAGTTTCTTGTGATCTTCC	103	XM_021162957.1
Notch1	F:GATGGCCTCAATGGGTACAAG R:TCGTTGTTGTTGATGTCACAGT	74	XM_021181098.1
Notch2	F:ATGTGGACGAGTGTCTGTTGC R:GGAAGCATAGGCACAGTCATC	146	XM_021157432.1
VEGF	F:CTGGATATGTTTGACTGCTGTGGA R:GTTTCTGGAAGTGAGCCAATGTG	180	NM_001110268.1
β-Catenin	F:CGCAAGAGCAAGTAGCTGATATTG R:CGGACCCTCTGAGCCCTAGT	61	XM_021171279.1
β-Actin	F:GGCTGTATTCCCCTCCATCG R:CCAGTTGGTAACAATGCCATGT	154	NM_007393.5

TNF-α, tumor necrosis factor-alpha; Icos, inducible costimulatory; SHH, Sonic Hedgehog; FGF7, fibroblast growth factor 7; VEGF, vascular endothelial growth factor.

### Statistical Analysis

Each experiment was repeated at least three times. The data were statistically analyzed using one-way ANOVA and the least squares difference test for the comparison among groups using SPSS 20. Significant differences were defined at p values of <0.05.

## Results

### Jagged1 and EGF Additively Enhanced Hair Follicle Development and Hair Growth

**Figure 1:**
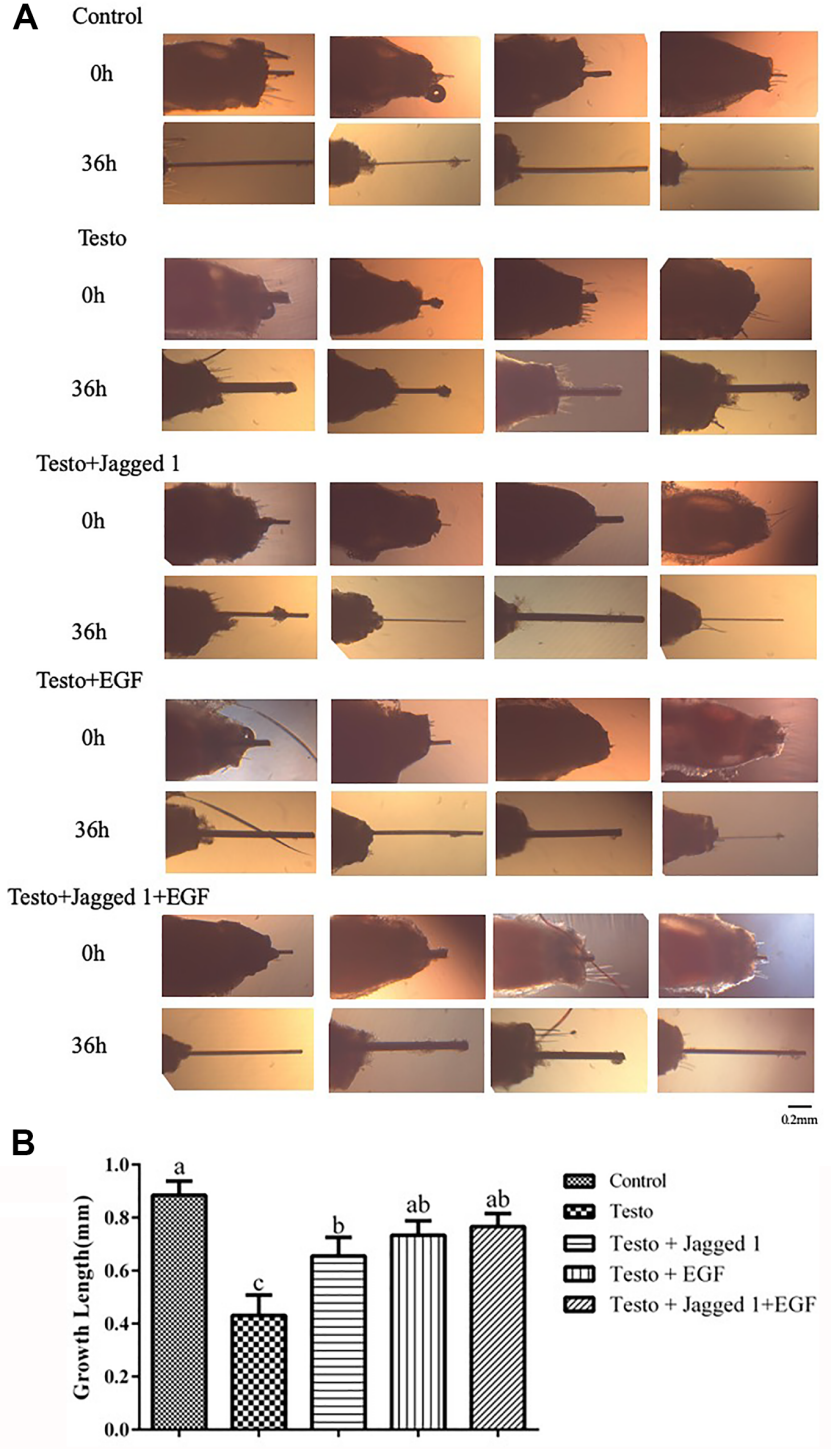
Effects of Jagged1 and/or EGF on mouse hair follicle growth *in vitro*. **(A)**. Images of hair follicles cultured in the absence and presence of Testo, Jagged1, and/or EGF at 0 and 36 hours of treatments, respectively. **(B)**. Quantitation results of hair growth *in vitro*. Data represent the mean ± SEM of from four hair follicles. Different letters denote statistical differences between groups (p < 0.05). Control (medium); Testo (20 μg/ml); Jagged1 (20 μg/ml Testo + 40 μg/ml Jagged1); EGF (20 μg/ml Testo+1 ng/ml EGF), and Jagged1 + EGF (20 μg/ml Testo + 40 μg/ml Jagged 1 + 1 ng/ml EGF).

The effect of Jagged1 and/or EGF on testosterone-suppressed hair growth *in vitro* was investigated. As shown in **Figure 1**, hair growth was significantly suppressed by testosterone. Addition of Jagged1 partially revered the inhibition effect testosterone, although not to the level of control. EGF, and EGF plus Jagged1 both revered testosterone inhibition completely (**Figure 1**).

We then further examined the *in vivo* effect of the two factors *in vivo*. To achieve synchronized anagen development over the back skin area, 50 mice were depilated at the dorsal back area, and randomly divided into five groups with the following topical treatments in the same volume: control (30% ethanol); testosterone to induce androgenetic alopecia (negative control; 0.5% testosterone (Patel et al., 2014); Jagged1 (0.5% testosterone + 1 mg/ml Jagged1); EGF (0.5% testosterone + 30 μg/ml EGF); and Jagged1 + EGF (0.5% testosterone + 1 mg/ml Jagged1 + 30 μg/ml EGF).

**Figure 2:**
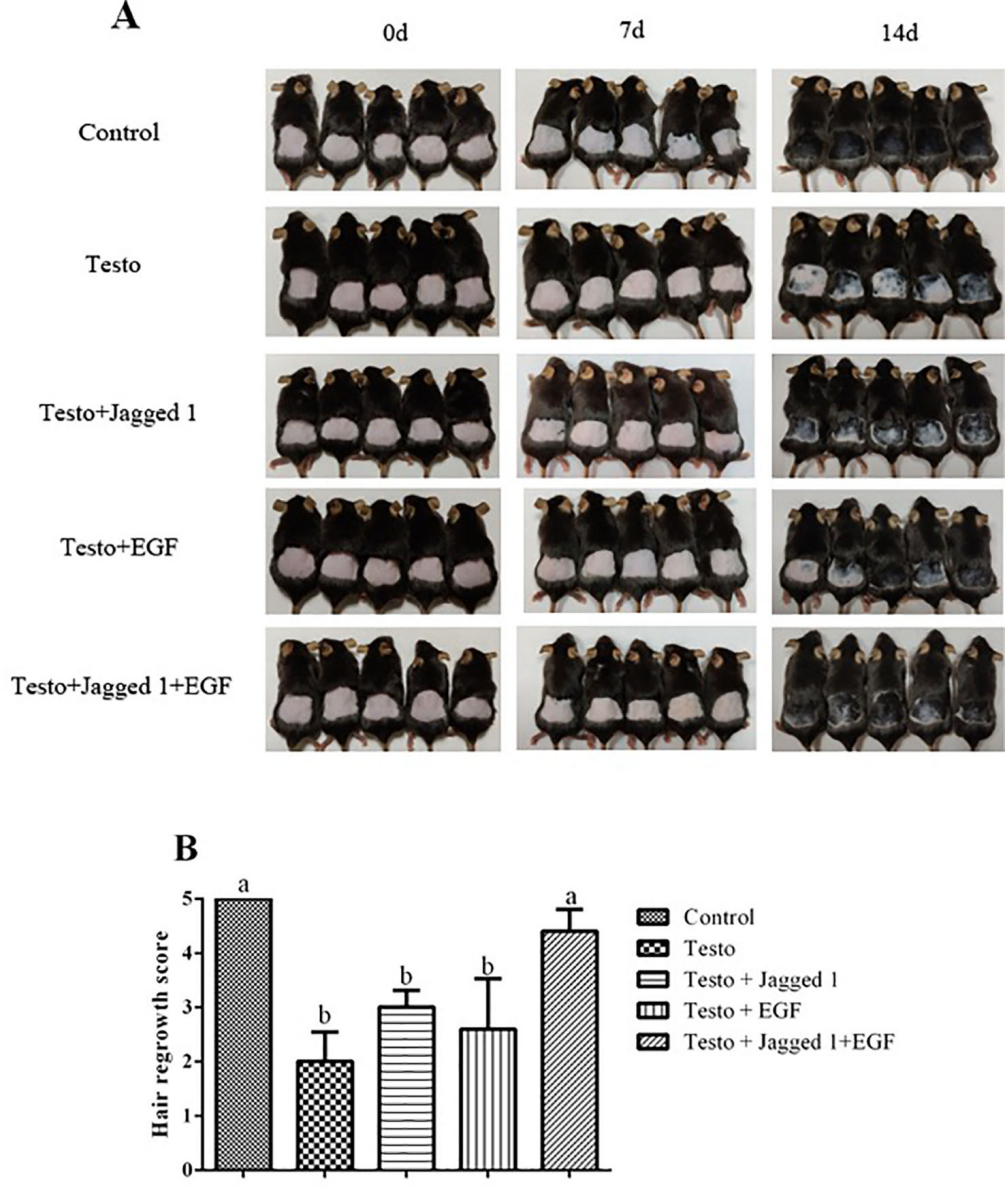
Influence of Jagged1 and/or EGF on hair growth. Mice were treated for 14 days with the indicated treatments, after which the mice hair regrowth were recorded. **(A)** Images of hair growth at day 0, 7, and 14, respectively. **(B)** Hair growth score. Data represent the mean ± SEM of five animals. Different letters denote statistical differences between groups (p < 0.05).

These treatments were applied twice a day for 14 days, and hair regrowth was assessed. As showed in **Figure 2**, testosterone (testo) significantly suppressed hair growth compared to control. The suppression was partially reversed by topical application of Jagged1 and EGF, respectively. In the presence of both Jagged1 and EGF, the hair growth stimulating effect was stronger than that of Jagged1 or EGF alone. Hair growth was further evaluated using the hair grow scoring system (Varghese et al., 2014). It was found that Jagged1 and EGF tended to partially reverse testosterone-suppressed hair growth, however, the revision was not significant statistically. In the presence of both the bioactive factors (Jagged1 + EGF), the hair growth score was increased to the level that was significantly higher than that of the testo group (p < 0.05), and not different from the non-testosterone positive control (**Figure 2B**).

**Figure 3:**
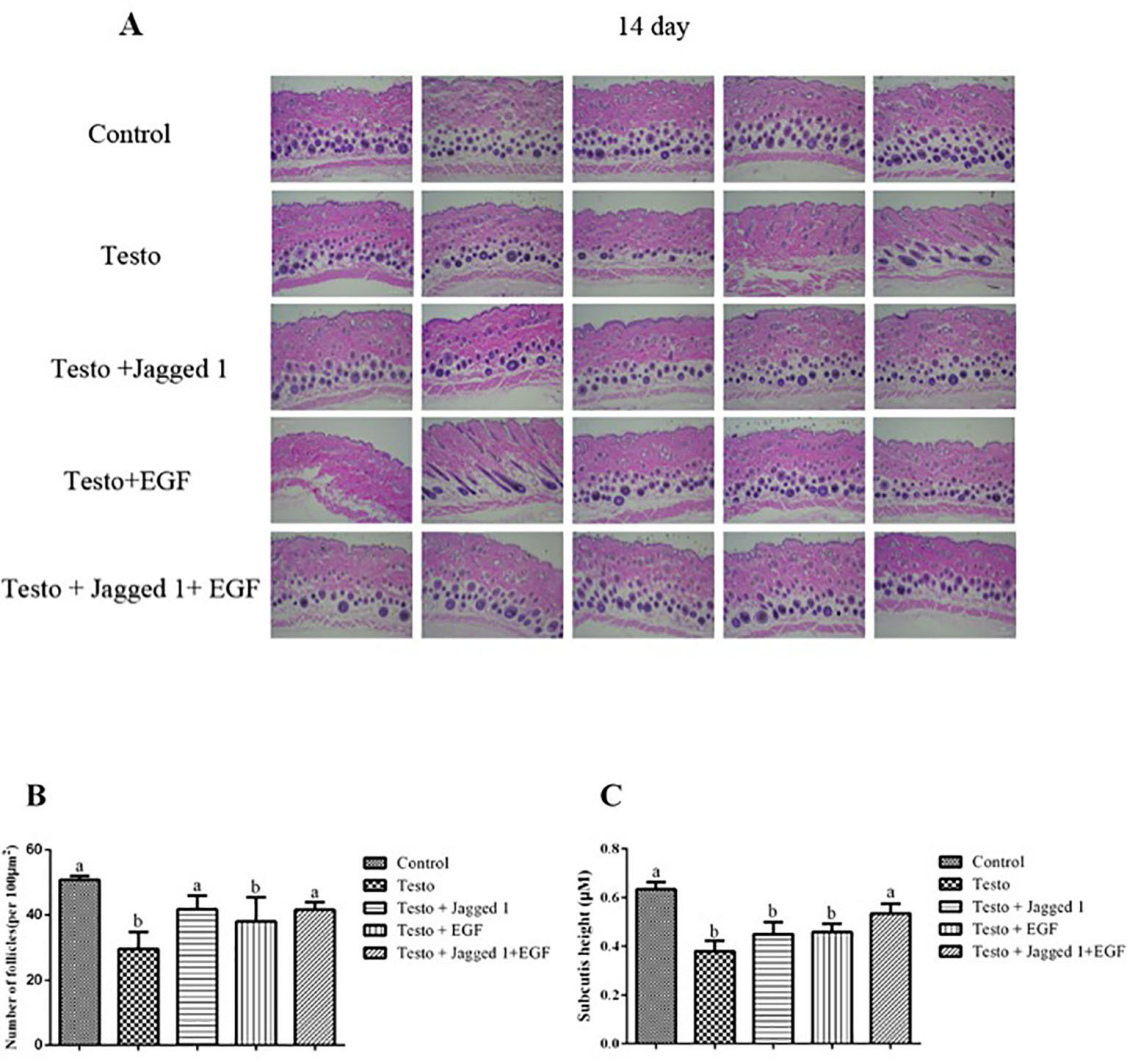
Influence of Jagged1 and/or EGF on hair growth. Mice were treated for 14 days with the indicated treatments and then euthanized. **(A)** Dorsal skin histological section images of histological section with H&E staining are shown. **(B)** Differential effects of Jagged1, EGF, and both on the number of follicles (per 100 μm^2^) at day 14. **(C)** Differential effects of Jagged1, EGF, and both on the height of subcutis area at day 14. Data represent the mean ± SEM of from five animals. Different letters denote statistical differences between groups (p < 0.05).

Histologically, consistent with what was observed in the hair growth in general, at day 14 of treatment the overall hair follicle appeared to be better developed in the following order: control > Jagged1 + EGF > Jagged1 > EGF > Testo group (**Figure 3A**). To better quantitate the histological response to treatment, follicle number was counted. As to be expected, significantly less hair follicle number was found in the testosterone treated group compared to the control (p < 0.05). Although there is a trend of follicle number increase in the EGF group, it is however, still not statistically significantly different than that of the testo group alone (**Figure 3B**). The follicle number decreased by testosterone was reversed to the level that it was not significantly different to that of non-testosterone control in the Jagged1, and Jagged1 + EGF groups (**Figure 3B**). When the height of the subcutis area was also measured, it was found that the thickness of subcutis decreased significantly by testosterone. While this decrease was partially, but not significantly reversed by Jagged1 and EGF, respectively, a significant reversion was observed in the presence of both Jagged1, and EGF (**Figure 3C**), suggesting more follicles are in the anagen stage in the presence of both of the bioactive factors.

### Effect of Jagged1 and/or EGF on Cell Proliferation and Apoptosis in Hair Follicles

**Figure 4:**
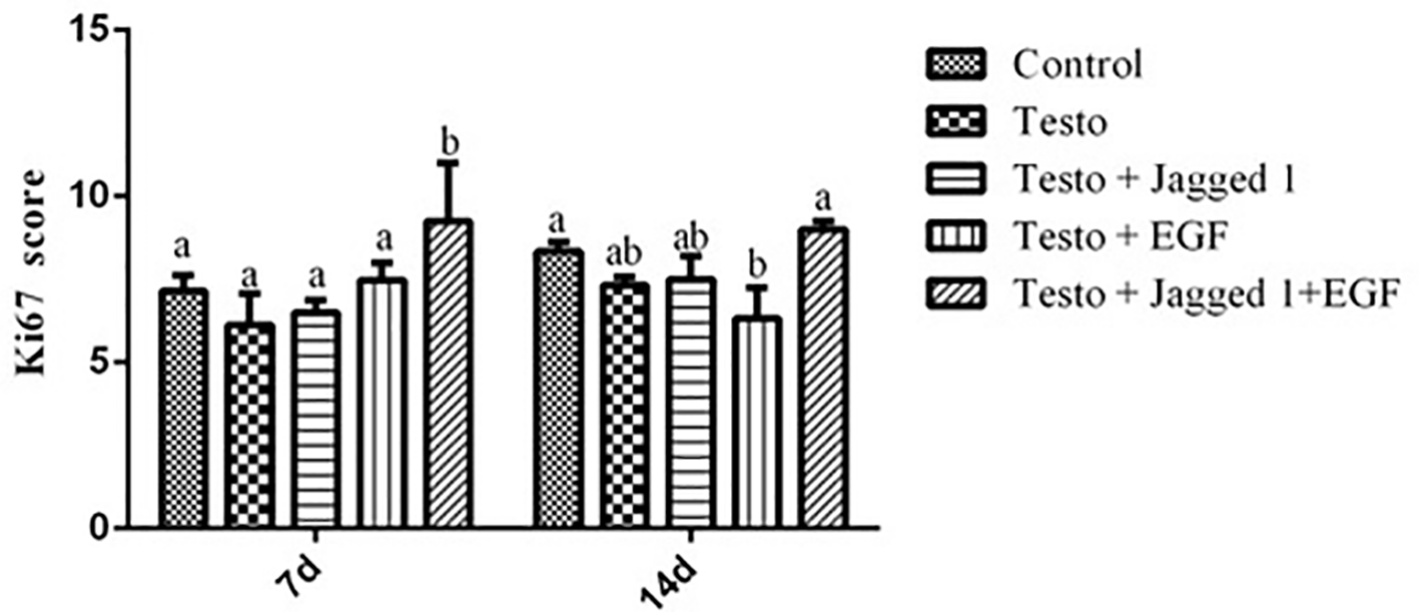
The effects of Jagged1 and/or EGF on Ki67 protein expression of Ki67 in mouse skin tissue *in vivo*. At day 7 and day 14 of treatments indicated, animals were euthanized, and immunohistochemistry was performed and analyzed using Open Source Plugin. Data represent the mean ± SEM from five animals. Different letters denote statistical differences between groups (p < 0.05).

Ki67 is expressed in the G1, S, G2, and M phase of the cell cycle, but not in resting cells in G0. Ki67 is thus regarded as proliferation marker (Lopez et al., 1991). To study if the improved hair growth may be associated with increased proliferation of hair follicle cell by Jagged1 and EGF, immunohistochemistry was performed using antibody against Ki67. At day 7 of treatment, while no increase of Ki67 positive score was observed in the Jagged1 and EGF group, respectively, the Ki67 staining score is the highest in the Jagged1 + EGF group, compared to that of the other four groups, suggesting that follicle cell proliferation was significantly enhanced when both of the bioactive factor are presence (**Figure 4**). By day 14, the Ki67 score in the Jagged1 + EGF is the same as that of the control group. Interesting, the score is lower in the EGF group compared to that of the control and Jagged1 + EGF groups at this time point (**Figure 4**).

**Figure 5:**
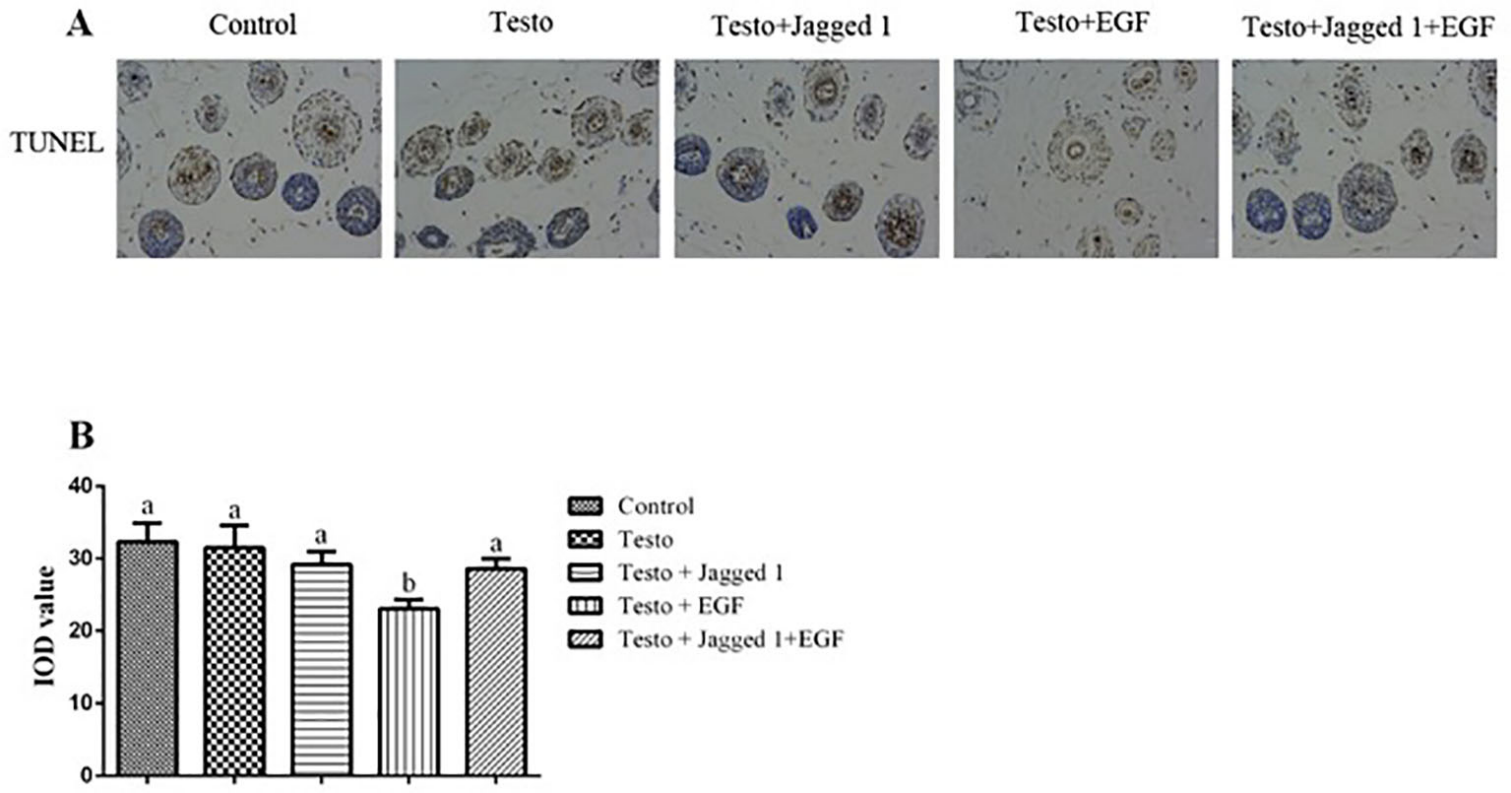
Influence of Jagged1 and/or EGF on apoptosis in mouse hair follicle. At day 14 of treatments, animals were euthanized, and terminal deoxynucleotidyl transferase dUTP nick end labeling (*TUNEL*) was performed, and data were analyzed using Image-Pro Plus 6.0 software. **(A)** representative images; **(B)** quantitation data. Data represent the mean ± SEM of results from three animals. Different letters denote statistical differences between groups (p < 0.05). Original magnification: 400×; IOD: Integrated optical density.

To study if Jagged1 and/or EGF may have an effect on apoptosis of the hair follicle cell, TUNEL analysis was perform. **Figure 5A** showed the representative images of TUNEL staining. Quantitation of apoptosis rate in hair follicle using Image-pro plus 6.0 software revealed that while EGF by itself suppressed apoptosis in hair follicle cells (p < 0.05), there was no difference in apoptosis rate among control, Testo, Jagged1, and Jagged1 + EGF groups (**Figure 5B**).

### Jagged1 and EGF Regulate Expression of Genes That Are Associated With Cell Proliferation, Differentiation, and Inflammation

**Figure 6:**
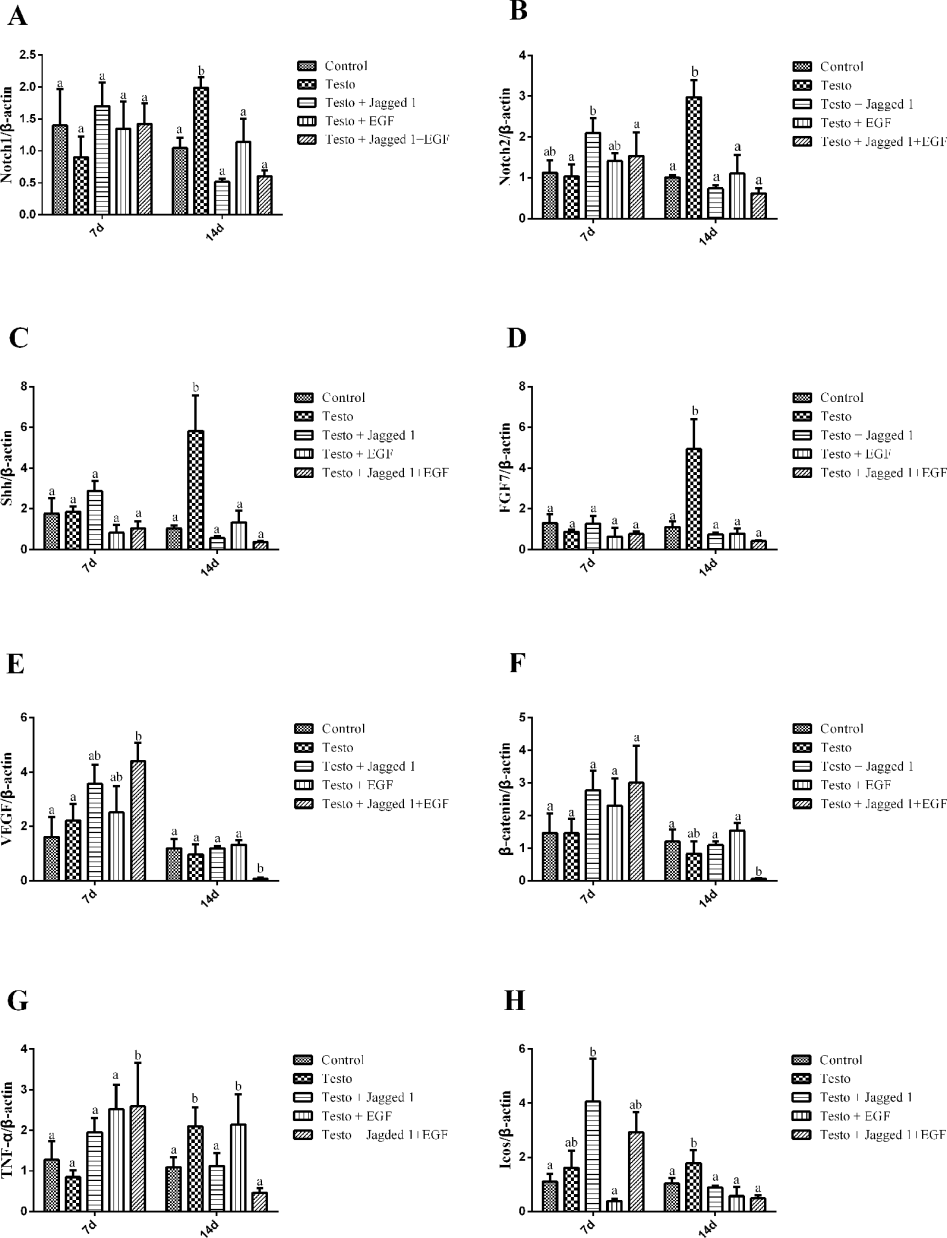
Jagged1 and/or EGF regulates the expression of Notch1, Notch2, Shh, FGF7, VEGF, and beta-catenin *in vivo*. RNAs were isolated from the back skin of C57BL/6 mice after 7 and 14 days of treatments as indicated. RT-PCR were performed. Data represent the mean ± SEM of results from three animals. Different letters denote statistical differences between groups (p < 0.05). **(A**-**E)** Jagged1 and/or EGF regulates the hair growth associated genes expression.

As Jagged1 and EGF promoted hair follicle growth, we next sought to study if they regulate the expression of genes such as Notch1, Notch2, Sonic hedgehog (Shh), keratinocyte growth factor (FGF7), vascular endothelial growth factor (VEGF), and β-catenin, which are associated with follicle cell proliferation and differentiation. RNAs were isolated from back skin of mice from days 7 and 14 treatment, and RT-PCR was performed. As shown in **Figure 5**, while Notch2 expression was increased by Jagged1 at day 7, its expression in other groups was unchanged. At day 14, testosterone increased Notch1 and Notch2 expression, and this induction was suppressed by Jagged1 and EGF, respectively and in combination to the level of control (**Figures 6A, B**). At day 7, no change was observed on the expression of Shh and FGF7 across various groups. At day 14, testosterone-induced Shh and FGF7 expression, and this induction was suppressed by Jagged1, EGF, and the combination of the two factors (**Figures 6C, D**). The expression of VEGF was increased in the Jagged1 + EGF group at day 7, although its mRNA level was down regulated in the Jagged1 + EGF group at day 14 (**Figure 6E**). Similar expression pattern was also observed for the expression of β-catenin (**Figure 6F**).

**Figure 7:**
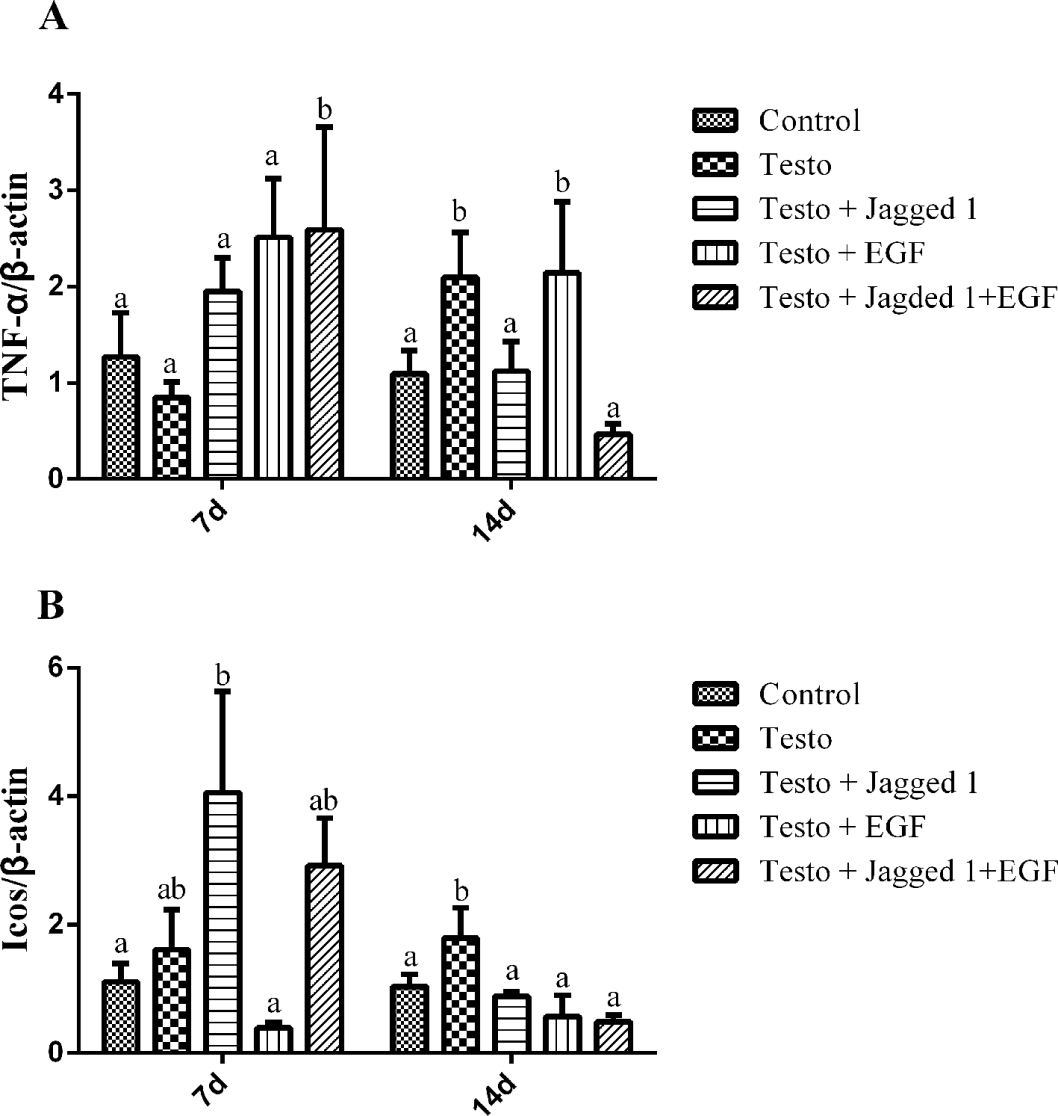
Regulation of Jagged1 and/or EGF on the expression of immune regulators, TNF-α, and Icos RNA were isolated from the back skin of C57BL/6 mice after 7 and 14 days of treatments as indicated. RT-PCR were performed. Data represent the mean ± SEM of results from three animals. Different letters denote statistical differences between groups (p < 0.05). **(A**, **B)** Jagged1 and /or EGF regulates the expression of immune regulator genes.

It is known that inflammation plays an important role in androgenetic alopecia (Trueb, 2002). To study if the hair growth promoting effect of Jagged1 and EGF may be associated with their involvement in inflammation modulation, we examined the expression of tumor necrosis factor alpha (TNF-α) and inducible T-cell costimulatory (Icos), which are both are known to be immunomodulators. TNF-α expression was increase in the Jagged1 + EGF group at day 7. By day 14, testosterone-induced TNF-α expression, and this induction was suppressed in the Jagged1 and Jagged1 + EGF groups to the level that is the same as the non-testosterone control (**Figure 7A**). Jagged1 increase Icos expression at day 7. However, by day 14, testosterone-induced Icos expression, and this induction was suppressed by Jagged1, EGF, and their combination to the level that is the same as the non-testosterone control (**Figure 7B**).

## Discussion

As to be expected, our result showed testosterone significantly decreases hair follicle number and the subcutis height. This finding is consistent with the report that testosterone causes a prolonged telogen phase and represses hair growth in the androgenetic alopecia rodent model (Park et al., 2003). The hair follicle density increased in the EGF and Jagged1 + EGF groups, demonstrating their effect on hair follicle growth. The finding that the height of the subcutis area increased in the Jagged1 + EGF group suggested that in the presence of both of the bioactive factors, more hair follicles are at the anagen growth phase.

In mammals, communication between epithelial cells and immune cells is a well-coordinated process to maintain tissue homeostasis and restore normal function after being stressed and reviewed in (Carrasco et al., 2019; Sequeira and Watt, 2019). Skin is one of the largest organs that has large amounts of immune cells including the T-reg cell, which is known to have an physiologically intimate interaction with hair follicle and secrete Jagged1 (Ali et al., 2017). Previous work using an inducible T-reg cell depleted model resulted in a lack of hair growth which was rescued by addition of exogenous Jagged1 (Ali et al., 2017). Our findings on Jagged1 partially stimulated androgen-suppressed hair growth confirmed this previous finding and is consistent with the role of Jagged1 in hair follicle development. These results suggest the therapeutic potential of this peptide. It is possible that Jagged1 activated HFSC and thus reversed androgen’s suppression on hair follicle growth.

While Jagged1 stimulates the proliferation and differentiation of hair follicle stem cells, EGF promoted the growth and migration of outer root sheath (ORS) cells of hair follicle (Zhang et al., 2016b). It was suggested that Jagged1 promotes follicle growth *via* induction of anagen (Ali et al., 2017). On the other hand, EGF is known to induce catagen-like effects on hair follicles (Paus, 1998; Paus and Cotsarelis, 1999; Chon et al., 2012; Pletz et al., 2012). Our finding that the hair growth stimulation is the best in the presence of both Jagged1 and EGF suggests a possible positive interaction, and additive effect of the two compounds. It is tempting to speculate that this additive effect may have been due to first anagen (by Jagged1) and then catagen (by EGF) being induced in sequence in the presence of these two bioactive peptides. Interestingly, Jagged1 and EGF alone are partially effective, but the androgen (testosterone) suppression of the hair growth score, and subcutis height were completely reversed only when both of the Jagged1 and EGF were present.

EGF treatment showed significant reduction in chemotherapy-induced alopecia, where it decreased the chemotherapy-induced apoptosis of keratinocytes in hair matrix and retarded the progression of chemotherapy-induced alopecia (Paik et al., 2013). Our finding that EGF-suppressed androgen apoptosis is indeed in agreement with this notion.

Wnt, Shh, and Notch pathways interplay between epithelial and mesenchymal cells determines hair follicle morphogenesis (Rishikaysh et al., 2014). Wnt/β-catenin pathways play an essential role in hair growth *via* anagen induction. β-Catenin, the transducer of Wnt signaling, induces transition of the hair growth cycle from the telogen to anagen phases, and is critical for the development and growth of hair follicles (Huelsken et al., 2001; Bierie et al., 2003; Van Mater et al., 2003). Transient activation of β-catenin results in hair regrowth in mice, while ablation of β-catenin results in hair shortening and abnormal regeneration of hair in the dermal papilla of mouse hair follicles (Van Mater et al., 2003; Enshell-Seijffers et al., 2010). Shh, a secreted signaling molecule, is involved in morphogenesis and late stage differentiation. Shh plays an important role in adult hair development. In adult mice, Shh expression is upregulated in early anagen (Sato et al., 1999; Fuccillo et al., 2006). FGF7 is one of the hair follicle growth-inducing genes and is considered to be an important endogenous stimulator for hair follicle growth, development, and differentiation (Rosenquist and Martin, 1996). VEGF is a growth factor that stimulates vasculogenesis and angiogenesis, stimulating hair growth by facilitating the supply of nutrients to the hair follicle, providing an increase in the base of the follicle diameter (Yano et al., 2001). Notch signaling pathway is involved in a variety of cell-fate decisions. The expression of Notch molecules in hair follicles is correlated with follicular differentiation (Kopan and Weintraub, 1993; Powell et al., 1998). The activation of Notch signaling is a prerequisite for initiation of hair follicle development (Xavier et al., 2013). Jagged1 is a well-known ligand and activator of the Notch pathway. The stimulation of Notch2 expression by Jagged1 at day 7 may be one of the paths for Jagged1 to up-regulate the Notch pathway. By day 14, testo-induced Notch1 and Notch2 expression was significantly down-regulated by Jagged1, EGF, and their combination. The mechanism for Jagged1 and EGF to suppress the androgen-induced Notch1 and Notch2 expression is currently unclear. It is possible that hair follicle growth is well underway in the presence of these bioactive factors at this time point, and that high level expression of the Notch is no longer required. Similar explanations may also be attributed to the expression pattern of Shh and FGF7 at day 14.

The increased β-catenin and VEGF expression at day 7 in the Jagged1 + EGF group suggests that both of the bioactive factors are required to up-regulate these hair growth regulators. The finding that the combined treatment decreased β-catenin expression at day 14 was surprising, as this is not consistent with the increased anagen follicle and hair growth by the treatments. It is tempting to speculate that the high expression of β-catenin and VEGF are not required as the hair were well re-grown at this time point in this group. However, future study with extended treatment length is required to reveal if the β-catenin level would return to normal levels to ensure continued hair growth.

It was reported that Jagged1 promotes leukocyte infiltration and angiogenesis (Su et al., 2016). Our finding that Jagged1 increased expression of inflammatory genes either by itself (on Icos) or in combination with EGF (TNF-α) at day 7 is consistent with this notion. Testosterone-induced expression of these inflammatory genes occurred until day 14, but their expression remained the same as that of the control in the presence of Jagged1 and/or EGF, suggesting that the androgen-induced inflammatory response has been overcome by these two bioactive peptides at this time point.

Our study showed that topical application of Jagged1 promoted hair regeneration in androgenetic alopecia in a mouse model. The effect of Jagged1 was enhanced when it was used in combination with EGF. Their hair growth promoting effect may be *via* their stimulation on cell proliferation and modulating the expression of an array of genes involved in hair follicle growth and differentiation. Our results provide insights into the therapeutic potential of these peptides for androgenetic alopecia.

## Data Availability Statement

The datasets generated for this study are available on request to the corresponding author.

## Ethics Statement

The animal study was reviewed and approved by the Foshan University Institutional Animal Care and Use Committee (FSUeae2018014).

## Author Contributions

YL: Performing experiments, data acquisition and analysis, interpretation, co-writing of manuscript. CL: Data analysis and interpretations, co-writing the manuscript. XZ: Performing experiments, data acquisition and analysis. BW: Discussion and critical revision of manuscript. JL: Project design, data interpretations, co-writing the manuscript. KL: Discussion and critical revision of manuscript.

## Conflict of Interest

The authors declare that the research was conducted in the absence of any commercial or financial relationships that could be construed as a potential conflict of interest.
